# 4,6,8-Triarylquinoline-3-carbaldehyde Derivatives: Synthesis and Photophysical Properties

**DOI:** 10.3390/molecules181215769

**Published:** 2013-12-17

**Authors:** Malose Jack Mphahlele, Adewale Olufunsho Adeloye

**Affiliations:** Department of Chemistry, College of Science, Engineering and Technology, University of South Africa, P.O. Box 392, Pretoria 0003, South Africa; E-Mail: adeloao@unisa.ac.za

**Keywords:** 6,8-dibromo-4-chloroquinoline-3-carbaldehyde, Suzuki-Miyaura cross-coupling, 4,6,8-triaryl/(triarylvinyl)quinoline-3-carbaldehydes, 4,6,8-triaryl-3-(4-fluorophenyl)amino)-*N*-(quinolin-3-yl)methylenes, 4,6,8-triarylquinoline-3-methanol, photophysical properties

## Abstract

Palladium catalyzed Suzuki-Miyaura cross-coupling of 6,8-dibromo-4-chloroquinoline-3-carbaldehyde with arylboronic and arylvinylboronic acid derivatives in the presence of potassium carbonate in aqueous dioxane afforded the corresponding 4,6,8-triarylquinoline-3-carbaldehydes, exclusively. These products were transformed into 4,6,8-triaryl-3-(4-fluorophenyl)amino)-*N*-(quinolin-3-yl)methylenes and their 4,6,8-triaryl-quinoline-3-methanol derivatives. The absorption and emission spectra were measured for the 4,6,8-triarylquinoline-3-carbaldehydes and their derivatives in selected solvents of different polarity.

## 1. Introduction

Polysubstituted quinolines in which the quinoline framework is linked to an aryl substituent directly or via a π-conjugated spacer to comprise a donor-π-acceptor system continue to attract considerable attention in synthesis because of their potential photophysical properties. Polyarylquinolines, for example, serve as emitting chromophores due to their inherent fluorescent properties [1,2,3]. Likewise, styrylquinolines prove to be dyes with enhanced photo- and electroluminescent properties and they find application in medicine and pharmacology [4,5,6,7]. The polyarylquinoline scaffold also serves as electron-acceptor unit in carbazole–quinoline and phenothiazine–quinoline copolymers and oligomers found to exhibit intramolecular charge transfer (ICT) [8]. Moreover, polyarylquinoline–based compounds constitute an important component in optoelectronic materials [9,10] and this moiety also constitutes a π-conjugated bridge in nonlinear optical polymers [11]. Similarly, quinolines are also interesting ligands for transition metal complexes for the development of organic electroluminescent diodes (OLEDs) [12,13]. More thermally stable and mechanically strong nonlinear optical polymers with donor-acceptor architecture incorporating quinoline unit as a π-conjugated bridge have been prepared before and their photophysical properties investigated [8,12]. The quinoline moiety acts only as an acceptor in the intramolecular charge transfer (ICT) [14], which occurs through-space interaction or orbital overlap between donor and acceptor groups [15,16]. This is because nitrogen atom (N-1) of the quinoline ring is more eletronegative than carbon and its lone pair electrons are in an orbital which is perpendicular to the π-ring system, which makes it difficult for N-1 to become a donor through inductive or resonance effects.

Our approach to polysubstituted quinolines bearing aryl or arylvinyl substituents involves the use of halogenoquinolines as substrates for metal catalyzed cross-couplings to incorporate the carbon-bearing substituents on the fused benzo ring and heterocyclic framework. Polyaryl- and polystyrylquinolines bearing substituents on the benzo ring would be difficult to prepare via the known methods such as the Skraup, Friedlander and the Doebner-von Miller reactions. A series of (*E*)-6-styrylquinoline-based compounds have been synthesized via the Wittig reaction of quinoline-3-carbaldehyde with and evaluated as novel imaging probes for β-amyloid (Aβ) plaques [17]. Access to facile and efficient methods for the synthesis halogenoquinolines coupled with their ease of structural elaboration via metal catalyzed cross-coupling reactions make it possible to realize simple novel fluorophores. In this investigation, we opted for the use of 6,8-dibromo-4-chloroquinoline-3-carbaldehyde as substrate for the Suzuki-Miyaura cross-coupling with aryl- and arylvinylboronic acids to yield the requisite donor-π-acceptor compounds for further chemical transformation. To understand the influence of substituents on intramolecular charge transfer (ICT), absorption and emission spectra of the 4,6,8-triarylquinoline-3-carbaldehydes and their derivatives were measured in selected solvents of different polarity.

## 2. Results and Discussion

**Scheme 1:**
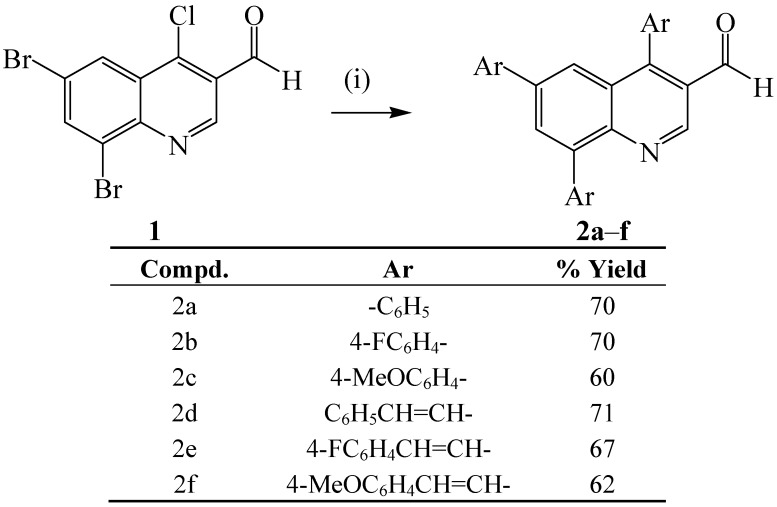
Synthesis of the 4,6,8-triarylquinoline-3-carbaldehydes **2a**–**f**.

We first reacted compound **1a** with phenylboronic acid (1 or 2 equiv.) in the presence of Pd(PPh_3_)_4_ or PdCl_2_(PPh_3_)_2_ as Pd(0) sources and potassium carbonate as a base in dioxane–water mixture (4:1, v/v) as a reference starting point for exploration of the coupling reactions based on literature precedents. In both cases the reaction led to the formation of an inseparable mixture of products. We opted for the use of an alkylphosphine ligand because these are known to coordinate with palladium and increase its electron density more so than arylphosphines and, in turn, accelerate the oxidative addition and reductive elimination steps in the catalytic cycle [18]. We then reacted **1a** with phenylboronic acid (1 or 2 equiv.) in the presence of dichlorobis(triphenylphosphine)palladium(II)-tricyclohexylphosphine (PdCl_2_(PPh_3_)_2_–PCy_3_) catalyst complex and potassium carbonate as a base in dioxane–water (4:1, v/v). In both cases, using 1 or 2 equiv. of phenylboronic acid, after 6 h we isolated along with the starting material, albeit in low yields (ca. 40%), a product characterized using a combination of spectroscopic techniques as 4,6,8-triphenylquinoline-3-carbaldehyde (**2a**). Although the Ar-Br bond is known to undergo oxidative addition with Pd(0) faster than Ar–Cl bonds [19], the observed lack of selectivity is presumably due to the fact that the 4-chloro atom on the quinoline ring is also highly activated towards metal-catalyzed cross-coupling reactions [20]. Moreover, the β-chloro-vinylcarbonyl nature of compound **1** leads to increased activation of the C4-Cl bond. Computed bond dissociation energies of the halogenated quinolines at B3LYP and G3B3 levels, on the other hand, also revealed that all of the positions on the fused benzo ring bearing identical halogen atoms have comparable C–X bond dissociation energies [21]. Likewise, dibromoquinolines such as 5,7-dibromo-quinoline [22], 8-benzyloxy-5,7-dibromoquinoline [23], 2-aryl-6,8-dibromo-4-methoxyquinolines [24] and 3,6,8-tribromoquinoline [25] were also found to undergo Suzuki cross-couplings with little or no selectivity to afford the corresponding polysubstituted derivatives. The Heck reaction of the analogous 6,8-dibromoflavone with methyl acrylate also afforded mixture of both 6- and 8-monosubstituted and 6,8-disubstituted flavones [26]. Lack of selectivity was also observed by Zhang *et al.* in their attempt to effect one-pot borylation of the analogous 8-bromo-6-chloroquinoline with bis(pinacolato)-diboron (1 equiv.), followed by Suzuki-Miyaura cross-coupling of the envisioned incipient quinoline-8-boronic acid with phenyl bromide [27]. To ensure exhaustive multiple coupling in cases where it is difficult or not possible to control the selectivity of cross coupling of halogenated quinoline derivatives, an excess of the arylboronic acid is often employed to drive the reaction to completion [22]. A similar strategy was also employed by Zhang *et al.* on 8-bromo-6-chloroquinoline with excess of bis(pinacolato)diboron (2.2 equiv.) and phenylbromide to afford 6,8-diphenylquinoline in 94% yield [27]. The observed lack of selectivity in the case of **1**, prompted us to make use of excess aryl/arylvinylboronic acids (3.5 equiv.) using dichlorobis(triphenylphosphine)palladium(II)–tricyclo-hexylphosphine (PdCl_2_(PPh_3_)_2_-PCy_3_) catalyst complex and potassium carbonate as a base in dioxane–water (4:1, v/v). We isolated by column chromatography on silica gel the 4,6,8-triarylquinoline-3-carbaldehydes **2a**–**f** as sole products (Scheme 1).

*Reagents and conditions*: (i) ArB(OH)_2_ (3.5 equiv.), PdCl_2_(PPh_3_)_2_, PCy_3_, K_2_CO_3_, dioxane–water (4:1, v/v), 90 °C, 3 h.

**Scheme 2:**
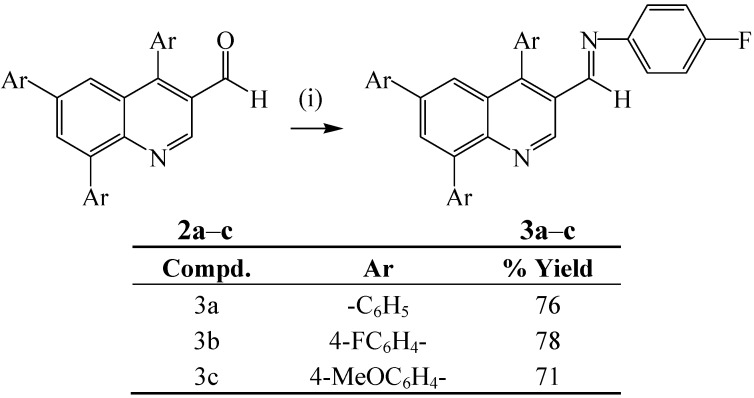
Synthesis of 4,6,8-triaryl-3-(4-fluorophenyl)amino)-*N*-(quinolin-3-yl)-methylenes **3a**–**c**.

With compounds **2a**–**f** in hand, we were ready to explore their reactivity in nucleophilic addition and reduction. The 4,6,8-triarylquinoline-3-carbaldehydes **2a**–**f** were subjected to nucleophilic addition with 4-fluoroaniline in dioxane under reflux (Scheme 2). We isolated the corresponding 4,6,8-triaryl-3-(4-fluorophenyl)amino)-*N*-(quinolin-3-yl)methylene derivatives **3a**–**c** from **2a**–**c**, with the styryl derivatives **2d**–**f** giving inseparable mixtures of products. The analogous 2/4-substituted quinoline-3-carbaldehydes and their hydrazine derivatives were previously found to exhibit *in vitro* activity against *Mycobacterium tuberculosis H37Rv* strain [28].

*Reagents and conditions*: (i) *p*-FC_6_H_4_NH_2_, dioxane, reflux, 12 h.

**Scheme 3:**
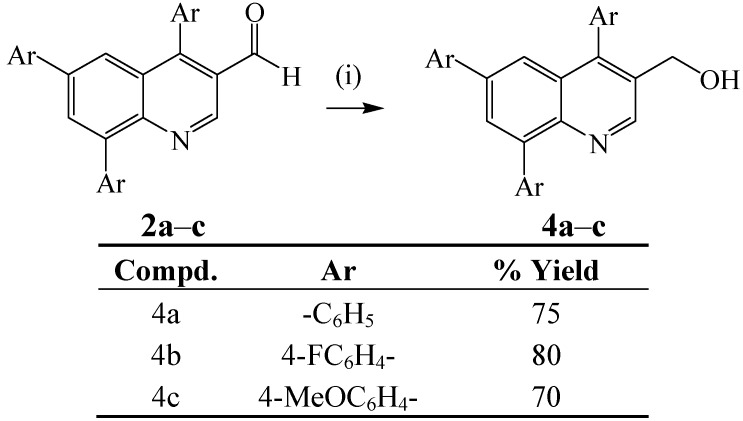
NaBH_4_-mediated reduction of carbaldehydes **2a**–**c** to alcohols.

Among the various reducible substrates, aldehydes are of great relevance in order to obtain the corresponding alcohols, which constitute important precursors to compounds with applications in the pharmaceutical and agricultural industries [29,30]. Since sodium borohydride has been employed to reduce aldehydes to primary alcohols [31], we reacted compounds **2a**–**c** with sodium borohydride in ethanol under reflux to afford the corresponding 4,6,8-triarylquinoline-3-methanol derivatives **4a**–**c** (Scheme 3).

*Reagents and conditions*: (i) NaBH_4_, EtOH, reflux, 0.5 h.

**Figure 1:**
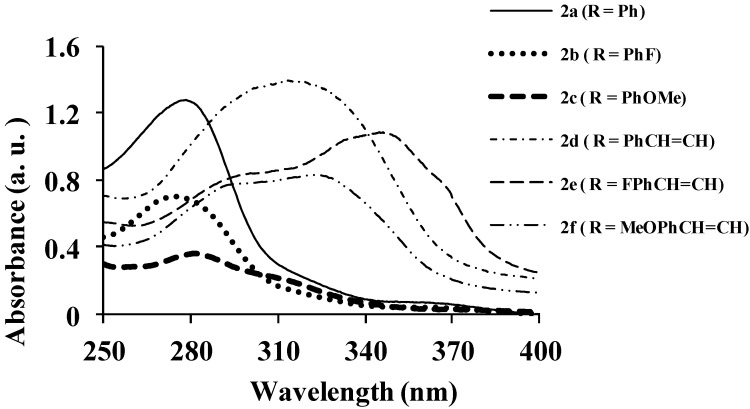
UV-Vis spectra of **2a**–**f** in CHCl_3_ (conc. 2.0 × 10^−7^ mol/L).

**Figure 2:**
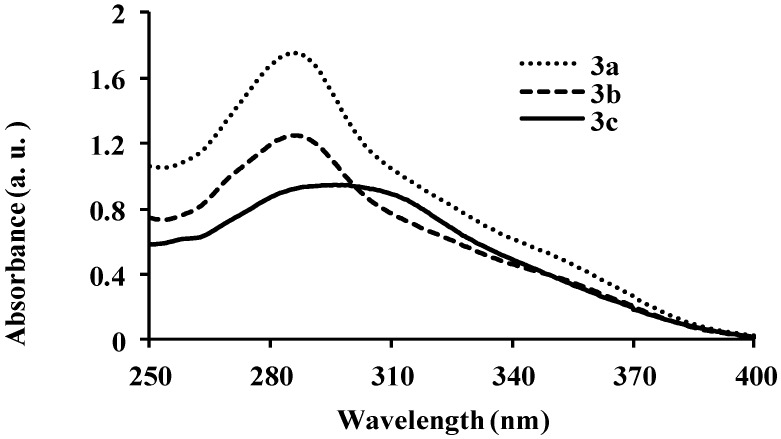
UV-Vis spectra of **3a**–**c** in CHCl_3_ (conc. 2.0 × 10^−7^ mol/L).

**Figure 3:**
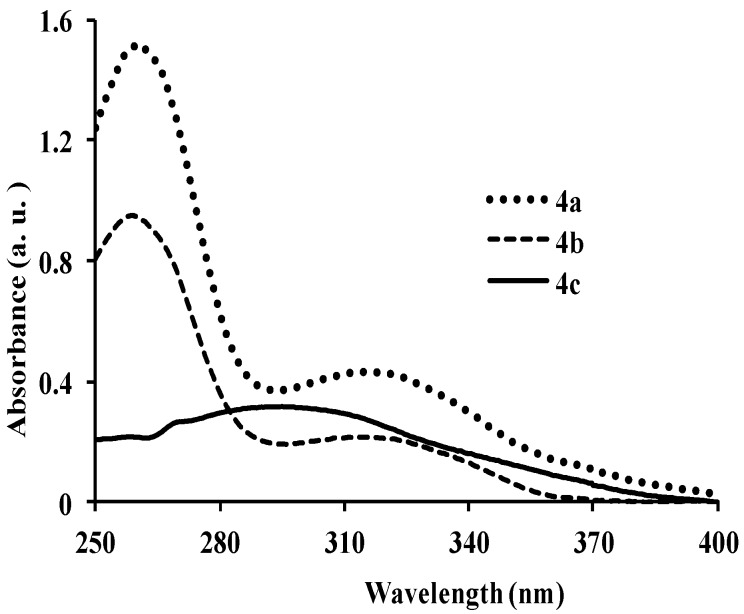
UV-Vis spectra of **4a**–**c** in CHCl_3_ (conc. 2.0 × 10^−7^ mol/L).

**Figure 4:**
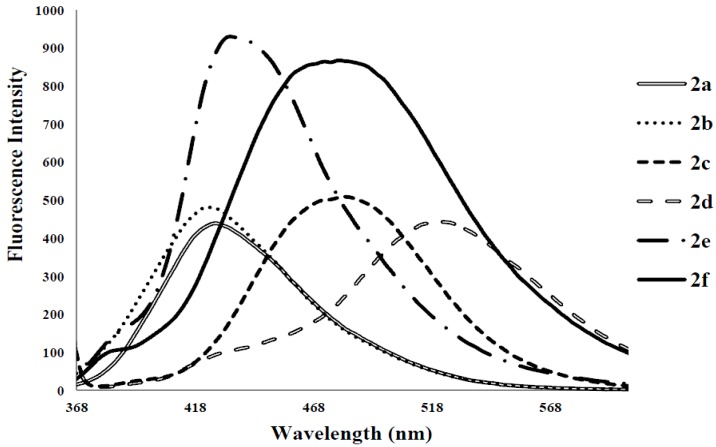
The fluorescence emission spectra of **2a**–**f** (λ_ex_ = 355nm) in CHCl_3_ (conc. 2.0 × 10^−7^ mol/L) at rt.

**Figure 5:**
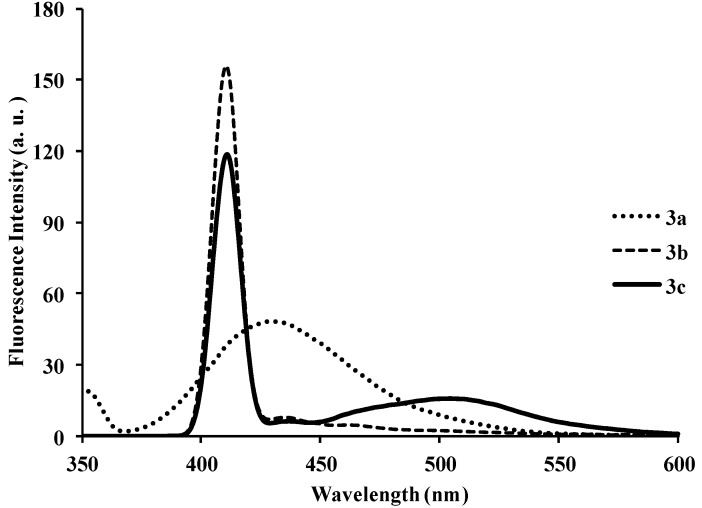
The fluorescence emission spectra of **3a**–**c** (λ_ex_ = 385 nm) in CHCl_3_ (conc. 2.0 × 10^−7^ mol/L) at rt.

**Figure 6:**
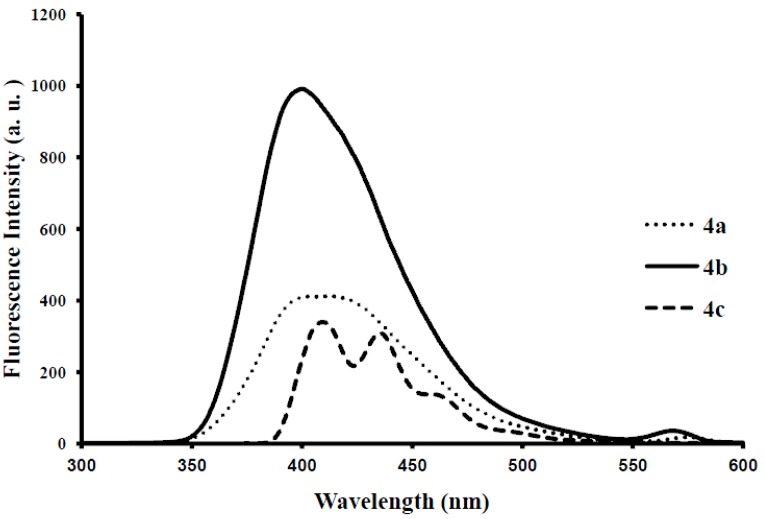
The fluorescence emission spectra of **4a**–**c** (λ_ex_ = 385 nm) in CHCl_3_ (conc. 2.0 × 10^−7^ mol/L) at rt.

**Figure 7:**
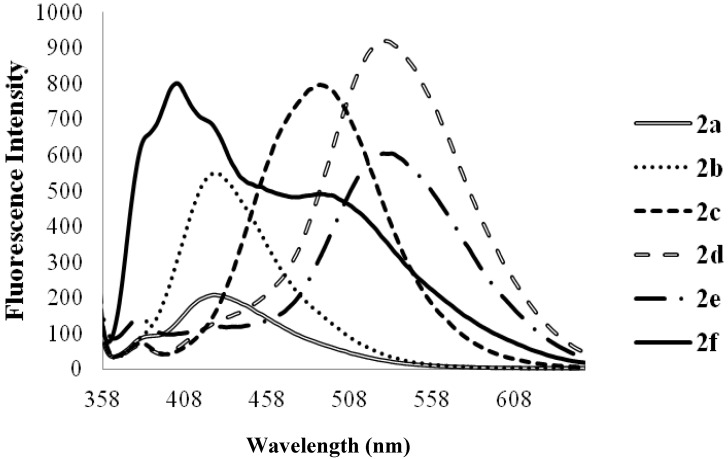
The fluorescence emission spectra of **2a**–**f** (λ_ex_ = 355 nm) in DMF, conc. (2.0 × 10^−7^ mol/L) at rt.

**Figure 8:**
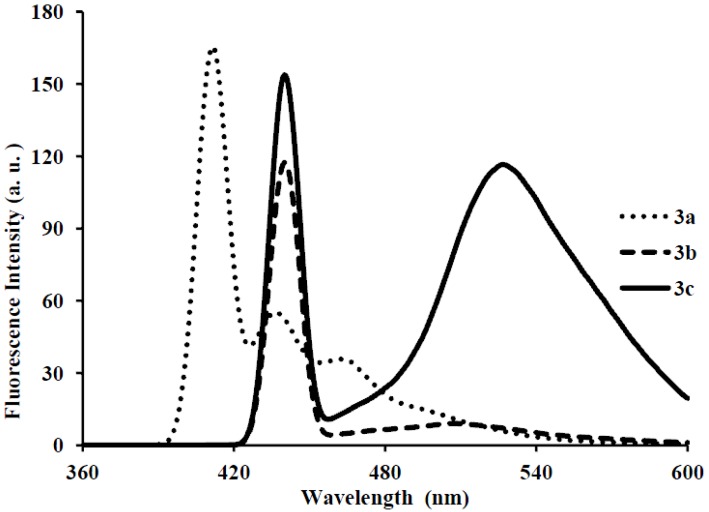
The fluorescence emission spectra of **3a**–**c** (λ_ex_ = 385 nm) in DMF (conc. 2.0 × 10^−7^ mol/L) at rt.

**Figure 9:**
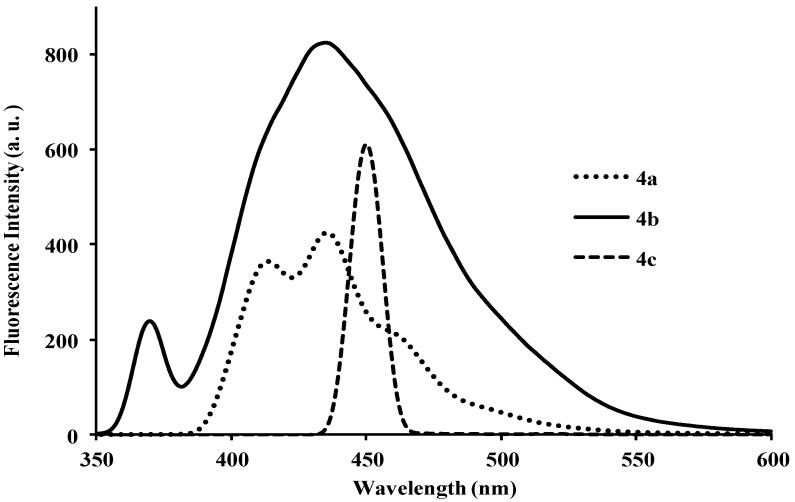
The fluorescence emission spectra of **4a**–**c** (λ_ex_ = 368 nm) in DMF (conc. 2.0 × 10^−7^ mol/L) at rt.

**Figure 10:**
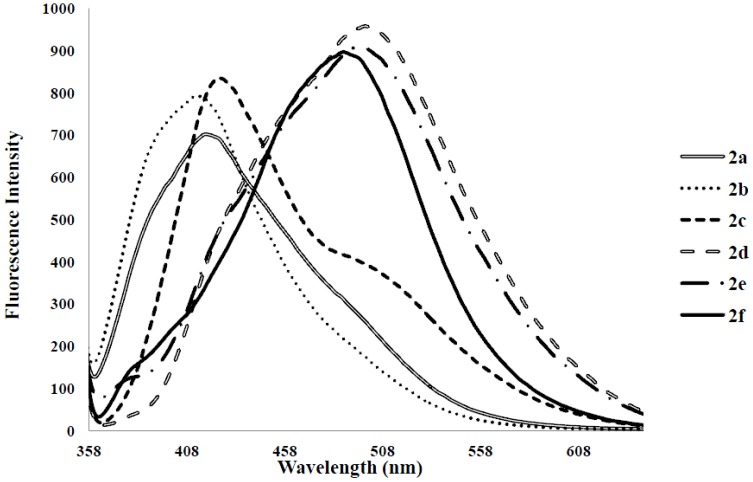
The fluorescence emission spectra of **2a**–**f** (λ_ex_ = 355 nm) in CHCl_3_–MeOH (1:1, v/v) (conc. 2.0 × 10^−7^ mol/L) at rt.

**Figure 11:**
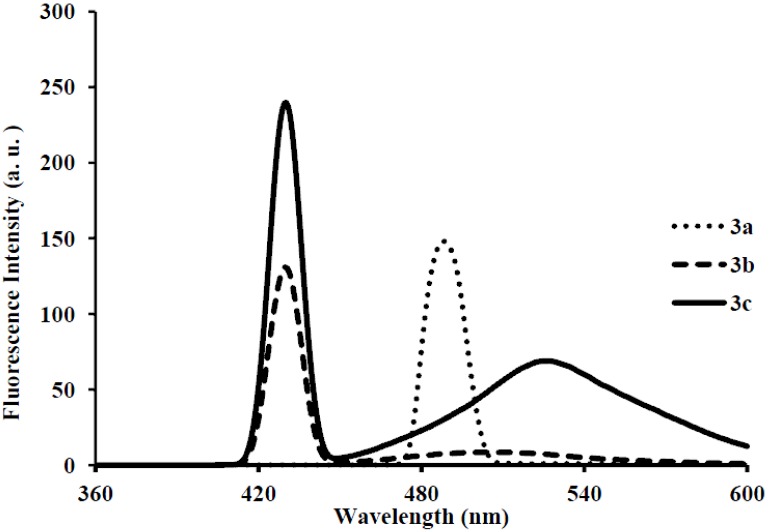
The fluorescence emission spectra of **3a**–**c** (λ_ex_ = 350 nm) in CHCl_3_–MeOH (1:1, v/v) (conc. 2.0 × 10^−7^ mol/L) at rt.

**Figure 12:**
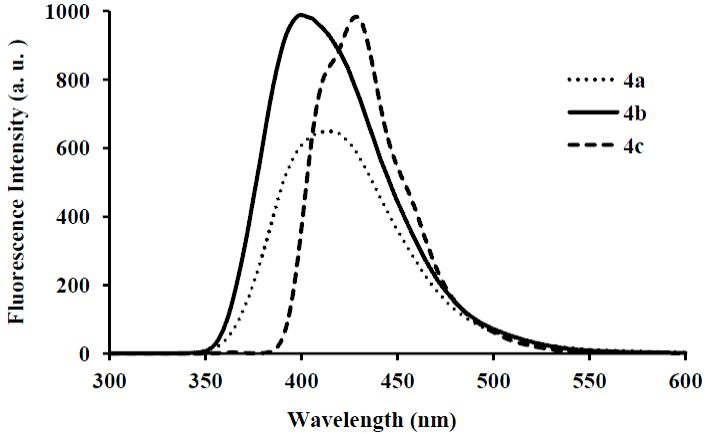
The fluorescence emission spectra of **4a**–**c** (λ_ex_ = 361 nm) in CHCl_3_–MeOH (1:1, v/v) (conc. 2.0 × 10^−7^ mol/L) at rt.

Compounds **2a**–**f** comprise the electron-deficient quinoline-3-carbaldehyde framework as an electron-acceptor linked to the aryl rings directly or via π-conjugated spacers to form donor-π-acceptor systems. As a prelude to compounds with potential photoelectronic properties, we determined the absorption properties of compounds **2a**–**f**, **3a**–**c** and **4a**–**c** in chloroform and their fluorescence properties in solvents of medium and high polarity (Figure 1, Figure 2, Figure 3, Figure 4, Figure 5, Figure 6, Figure 7, Figure 8, Figure 9, Figure 10, Figure 11, Figure 12 and Table 1). In the ultraviolet region, the 4,6,8-triarylquinoline-3-carbaldehydes **2a**–**c** in which the electron-deficient quinoline framework is linked directly to the aryl ring absorb at λ_abs_ 275 (**2b**), 278 (**2a**) and 282 nm (**2c**) due to the π–π* transitions of conjugated quinoline ring in analogy with the assignment for π-styrylquinolines [32] (Figure 1). Increased peak broadening and reduced intensity for **2c** are attributed to the strong electron donating effect of the methoxy groups which may interfere with the conjugation of the π electrons restricting the transition from bonding orbital to antibonding orbital. The presence of π-conjugated spacers in the styryl derivatives **2d**–**f**, on the other hand, leads to bathochromic shifts of the wavelengths at λ_abs_ 315 nm for **2d**, 323 nm for **2f** and 347 nm for **2e** with increased molar extinction coefficient as well as broadening of the absorption maxima. Figure 2 and Figure 3 correspond to the optical absorption spectra of compounds **3a**–**c** and **4a**–**c** in CHCl_3_, respectively. The amino-*N*-methylene substituted derivatives **3a** and **3b** exhibit relatively intense absorption bands at λ_abs_ 286 nm than **3c** with a slight red-shift and broadened band at λ_abs_ 295 nm (Figure 2). Compared with the corresponding 3-carbaldehyde precursors **3a**–**c** (Figure 1), compounds **3a**–**c** exhibit better molar absorbance and bathochromic shifts presumably due to the additional donor properties of the amino group leading to the lowering of the energy of the π*-orbital of the quinoline ring. Similar absorption patterns, however, with relatively less intense bands are observed for compounds **4a**–**c** (Figure 3). The blue-shift in wavelengths and the lower absorption intensity of **4a**–**c** (λ_abs_ *ca.* 22 nm and 35 nm) relative to compounds **2a**–**c** and **3a**–**c** may be the consequence of the reduction in π-conjugation brought by the saturated σ-bond of the methanol substitution on the quinoline scaffold. Compounds **4a** and **4b** exhibit additional lower intensity bands at λ_abs_ *ca.* 320 nm due to intermolecular charge transfer. The high energy absorption bands for compounds **2**–**4** lower than λ 380 nm are ascribed to the π–π* transitions [32]. 

**Table 1:** The absorption and emission data for compounds **2a**–**f**, **3a**–**c** **and** **4a**–**c**.

Compounds	λ_max_ (nm) CHCl_3_	(ε) × 10^4^ Mol^−1^cm^−1^	λ_em _(nm) CHCl_3_	λ_em _(nm) DMF	λ_em _(nm) CHCl_3_-CH_3_OH (1:1)	^(a)^ Quantum Yield (Φ)	Stokes Shift (CHCl_3_)
2a	278	3.63	424	425	408	0.07	146
2b	275	3.06	424	430	406	0.14	149
2c	282	1.70	474	483	415	0.28	192
2d	315	6.43	529	543	508	0.06	214
2e	347	5.99	420, 430	543	504	0.17	53, 73
2f	323	4.57	370, 470	405, 508	490	0.21	47, 147
3a	286	8.40	429	411	483	0.01	143
3b	286	6.64	429	440	430	0.03	143
3c	295	5.37	410	440, 527	430, 528	0.03	115
4a	260, 318	5.85	410	464	415	0.19	92, 150
4b	260, 320	0.92	399	372, 436	400	0.21	139, 79
4c	297	1.50	461	451	429	0.22	164

^(a) ^The relative quantum yields were calculated according to the equation indicated under Experimental section using quinine sulfate as the standard (Φ_q_ = 0.55) in 0.5 M H_2_SO_4_.

Photoluminescence characteristic properties of compounds **2a**–**f**, **3a**–**c** and **4a**–**c** were examined in chloroform, DMF and a mixture of chloroform–methanol (1:1, v/v) at room temperature as a meaningful means of extending investigation of electronic interactions of the 3-substituted 4,6,8-triarylquinoline scaffold in the excited state. At the excitation wavelength (λ_ex_ 355 nm) using quinine sulfate as standard, the fluorescence spectra of compounds **2a**–**c** in chloroform at room temperature show similar patterns and are characterized by single broad emission bands at λ_em_ 424 nm for **2a** and **2b** and at λ_em_ 474 nm for **2c** (Figure 4). The increased broadening and pronounced red band shift effect for **2c** are presumably due to increased π-electron delocalization of the 4-methoxyphenyl groups toward the electron-deficient quinoline ring. Emission wavelength bands at 529, 430 and 470 nm in the spectra of **2d**, **2e** and **2f** in chloroform are attributed to π-π* transition of the conjugated system and the corresponding less intense bands at λ 420 and 370 nm (**2e** and **2f**) are probably of charge transfer nature. The moderately and strongly donating fluoro and methoxy groups in compounds **2e** and **2f** are expected to increase π-electron delocalization into the quinoline ring leading to increased emission maxima. The styryl derivatives **2e** and **2f** gave exhibit strong fluorescence intensity in chloroform compared to compounds **2a**–**d**. The relative quantum yield values for compounds **2a**–**f** reflect the electronic effect of the aryl substituents (Table 1). The introduction of vinyl groups in **2d**–**f** caused large Stokes shift maxima in **2d**, however, with a resultant low quantum yield comparable to **2a**. Upon excitation at λ_ex_ 350 nm, compounds **3a**–**c** showed characteristic fluorescence emission at 429 nm for **3a**, **3b** and 410 nm for **3c** in chloroform (Figure 5). Excitation of compounds **4a**–**c** (Figure 6) at λ_ex_ 350 nm in chloroform resulted in significant high fluorescence emission intensity for the 4-fluorophenyl substituted derivative **4b** (λ_em_ 395 nm) than for derivatives bearing the phenyl (**4a**) (λ_em_ 410 nm) and 4-methoxyphenyl groups (**4c**), (λ_em_ 410 nm). Compounds **3a**–**c** which exhibit comparable Stokes shift with either **2a**–**c** or **4a**–**c** exhibit reduced fluorescence quantum yields due to the presence of the 3-(4-fluorophenyl)azomethine group (Table 1). The latter presumably decreases the electron density of the quinoline ring thus leading to reduced fluorescence quantum yields for **3a**–**c.**

Since the π,π* state is much more polarizable than the ground state, a change in polarity of the medium has been previously found to cause measurable displacements of the π-π* transition towards the red bands [33]. Less pronounced ICT and therefore reduced maxima for **2a** in DMF are presumably the result of reduced π-electron delocalization into the quinoline ring by the phenyl groups (Figure 7). This compound exhibits poor fluorescence properties in polar aprotic solvents. Relatively increased emission maxima observed in the spectra of compounds **2b** and **2c** are due to increased π-electron delocalization into the quinoline ring by the moderately and strongly donating 4-fluorophenyl and 4-methoxyphenyl groups. Increased fluorescent intensity and a shift to relatively longer wavelength observed for the styrylquinolines **2d** and **2e** are due to π-π* transition by the styryl groups. The styryl derivative **2d** exhibits a higher fluorescence intensity in DMF than the 4-substituted styryl derivatives **2e** and **2f**. The 4-methoxyphenyl bearing derivatives in which the aryl groups are linked directly (compound **2c**) to the quinoline framework or via vinyl bridge (compound **2f**) exhibit comparable fluorescence intensities, however, of different charge transfer nature. The two emission maxima observed for **2f** in DMF are probably due to competing through-space charge transfer and π-π* transition by the strongly electron-donating methoxystyryl groups. This additional red-shifted emission band of lower intensity may also be the result of the re-absorption of light emitted and/ or molecular excited state interaction with a ground state molecule [34]. Additional interaction of DMF with the methoxy group of **2f** would reduce its propensity for π-electron pair delocalization and such interaction will probably result in relatively less pronounced ICT due to π-π* transition. No appreciable difference in fluorescence intensity was observed for compounds **3a**–**c** in DMF except for the bathochromic shifts of the wavelengths of *ca.* 30 nm for **3b** and **3c**, and a blue-shift of *ca.* 18 nm for **3a** (Figure 7, Figure 8, Figure 9). Moreover, the emission at long wavelength for **3c** at 527 nm was significantly quenched in both **3a** and **3b**, mirroring the slight bathochromic shift observed in the electronic absorption spectra, which appears broadened. The additional undesired red-shifted emission band of reduced intensity exhibited by **3c** is presumably due to the re-absorption of light emitted and/or molecular excited state interaction with a ground state molecule leading to a partial transfer of charge in the molecule [34]. It is rather difficult to quantitatively explain the quenching of **4a** and **4c** in both chloroform and DMF. The possibility of the electronic interactions between the singlet excited states is not precluded. However, it should be noted that the electronic absorption spectrum of **4c** had a broad band with no significant absorption in the UV-region.

A strongly polar protic solvent such as methanol would interact with the carbonyl moiety and probably render the quinoline ring more electron deficient. Due to poor solubility in cold methanol, the fluorescence spectra of compounds **2a**–**f** were taken in a chloroform–methanol mixture (1/1, v/v). The emission spectra are characterized by intense emission maxima due to π-π* transition resulting from direct π-electron delocalization by the aryl groups for **2a**–**c** or along the vinylene bridges for **2d**–**f** (Figure 10). Polar protic methanol is expected to form strong intermolecular hydrogen bonds with the carbaldehyde group and/ or N-1 and, in turn, make the quinoline framework more electron-deficient. The increased intensities and bathochromic shifts for compounds **2d**–**f** relative to **2a**–**c** are presumably the result of increased π-electron delocalization along the vinylene bridges towards the electron-deficient quinoline ring. The solvent polarity dependent electronic transitions are attributed to efficient intramolecular charge transfer (ICT) processes, in which the HOMOs and LUMOs are localized on the styrene-based ring and the quinoline-based moiety, respectively. Literature precedent, on the other hand, reveals that if hydrogen bonding occurs in the donor, the tendency is for the absorption maximum to shift in the opposite direction [35]. The slight shifts in wavelengths observed for the 4-methoxyphenyl and 4-methoxystyryl derivatives **2c** and **2f** are presumably the consequence of additional hydrogen bonding between methanol and the methoxy substituents. In chloroform-methanol mixture (Figure 11), emission intensity and wavelength are significantly enhanced with emission wavelengths shifted to the red at *ca.* 54 nm in **3a** and 20 nm in **3b** and **3c**, respectively (Figure 11). Moreover, the emission at long wavelength at 527 nm for **3c** was significantly quenched in both **3a** and **3b** and this effect mirrors the slight bathochromic shift observed in the electronic absorption spectra, which appears broadened. Upon excitation at 385 nm in chloroform-methanol mixture, the characteristic fluorescence emissions of **3a**, **3b** and **3c** observed at 429 nm and 410 nm in chloroform (see Figure 5), are significantly enhanced in the solvent mixture with emission wavelengths shifted to the red *ca.* 54 nm for **3a** and 20 nm for **3b** and **3c**, respectively (Figure 11). However, the fluorescence intensity of compounds **3a**–**c** was found to be reduced, and as low as 50% when compared to the **2a**–**c** series (see Figure 5). There are some plausible reasons for the difference in the intensity of these compounds in the chosen solvents: (i) it may be associated with the compound/solvent interaction through the solvent’s optical parameters (refractive index and dielectric constant); (ii) it may be related to the magnitude of the change in dipole moment of the electronic ground state and the excited state and (iii) there may be some conformational changes induced by the solvent that modify the effective conjugation lengths of the emitting chromophores, most especially the presence of additional 4-fluorophenyl group on nitrogen [36,37,38].

When compounds **4a**–**c** were run in a mixture of chloroform-methanol at varied excitation wavelengths (λ_ex_ 347 nm) for **4a**, (λ_ex_ 339 nm) for **4b** and (λ_ex_ 361 nm) for **4c**, appreciable strong fluorescence intensities were observed in all the compounds (Figure 12). Compound **4c** bearing the strong electron donating 4-methoxyphenyl group exhibits relatively higher emission intensity at wavelength 429 nm as compared to compounds **4a** and **4b**. The observed red-shift in wavelength for **4c** is presumably the result of hydrogen bonding between methoxyphenyl groups and methanol.

## 3. Experimental

### 3.1. General

Melting points were recorded on a Thermocouple digital melting point apparatus and are uncorrected. IR spectra were recorded as powders using a Bruker VERTEX 70 FT-IR Spectrometer with a diamond ATR (attenuated total reflectance) accessory by using the thin-film method. The UV-vis spectra were recorded on a Cecil CE 9500 (9000 Series) UV-Vis spectrometer while emission spectra were taken using a Perkin Elmer LS 45 fluorescence spectrometer. The quantum efficiencies of fluorescence (Φ_fl_) were obtained with the following equation:

Φ_x_ = Φ_st_*(*F_x_*/*F_st_*)*(A_st_/A_x_)*(*n*_x_^2^/*n*_st_^2^)

*F* denotes the area under the fluorescence band (*F = *^a^*I*_fl_(λ), where *I*_fl_(λ) is the fluorescence intensity at each emission wavelength), A denotes the absorbance at the excitation wavelength, and *n* is the refractive index of the solvent [39]. For column chromatography, Merck Kieselgel 60 (0.063–0.200 mm) was used as stationary phase. NMR spectra were obtained as CDCl_3_ solutions using a Varian Mercury 300 MHz NMR spectrometer and the chemical shifts are quoted relative to the solvent peaks. Low- and high-resolution mass spectra were recorded at an ionization potential of 70 eV using a Micromass Autospec-TOF (double focusing high resolution) instrument. The synthesis and analytical data of **1** have been described before [24].

### 3.2. Suzuki-Miyaura Cross-Coupling of 1 with Arylboronic Acids. Typical Procedure

6,8-Dibromo-4-chloroquinoline-3-carbaldehyde (**1**, 1 equiv.), phenylboronic acid (3.5 equiv.), PdCl_2_(PPh_3_)_2_ (10% of **1**), PCy_3_ (20% of **1**) and K_2_CO_3_ (3 equiv.) in dioxane–water (4:1, 15 mL/mmol of **1**) were added to a two-necked flask equipped with a stirrer bar, rubber septum and a condenser. The mixture was flushed for 20 min with argon gas and a balloon filled with argon gas was connected to the top of the condenser. The mixture was heated with stirring at 80–90 °C under argon atmosphere for 3 h and then allowed to cool to room temperature. The cooled mixture was poured into ice-cold water and the product was taken-up into chloroform. The combined organic extracts were washed with brine, dried over anhydrous MgSO_4_, filtered and then evaporated under reduced pressure. The residue was purified by column chromatography to afford **2**. The following products were prepared in this fashion:

*4,6,8-Triphenylquinoline-3-carbaldehyde* (**2a**). Yellow solid (0.385 g, 58%), m.p. 213–215 °C (ethanol); *R*_f_ (10% ethyl acetate–hexane) 0.58; ν_max_ (ATR) 698, 781, 1381, 1443, 1480, 1568, 1685 cm^−1^; δ_H_ (CDCl_3_) 7.37–7.63 (m 13H), 7.74 (dd, *J =* 1.5 and 8.4 Hz, 2H), 7.85 (d, *J =* 2.1 Hz, 1H), 8.12 (d, *J =* 2.1 Hz, 1H), 9.44 (s, 1H), 9.95 (s, 1H); δ_C_ (CDCl_3_) 124.4, 125.4, 127.4, 127.5, 127.8, 128.0, 128.2, 128.7, 129.0, 129.3, 130.3, 130.7, 132.7, 132.8, 139.0, 139.9, 140.0, 141.9, 147.2, 147.7, 153.4, 191.8; *m/z* 386 (100, MH^+^); HRMS (ES): MH^+^, found 386.1540. C_28_H_20_NO^+^ requires 386.1545.

*4,6,8-Tris(4-fluorophenyl)quinoline-3-carbaldehyde* (**2b**). Yellow solid (0.440 g, 70%), m.p. 202–204 °C (ethanol); *R*_f_ (10% ethyl acetate–hexane) 0.59; ν_max_ (ATR) 827, 1159, 1223, 1486, 1511, 1600, 1685 cm^−1^; δ_H_ (CDCl_3_) 7.14 (t, *J =* 9.3 Hz, 2H), 7.22 (t, *J =* 9.3 Hz, 2H), 7.33 (t, *J =* 9.3 Hz, 2H), 7.47 (t, *J =* 9.3 Hz, 2H), 7.54 (t, *J =* 9.3 Hz, 2H), 7.70 (dt, *J =* 3.0 and 9.3 Hz, 2H), 7.74 (d, *J =* 1.2 Hz, 1H), 8.03 (d, *J =* 1.2 Hz, 1H), 9.41 (s, 1H), 9.96 (s, 1H); δ_C_ (CDCl_3_) 115.2 (d, ^2^*J*_CF_* =* 21.1 Hz), 116.1 (d, ^2^*J*_CF_ = 21.4 Hz), 116.1 (d, ^2^*J*_CF_ = 21.6 Hz), 124.0, 125.7, 127.5, 128.5 (d, ^4^*J*_CF_* =* 3.5 Hz), 129.1 (d, ^3^*J*_CF_* =* 8.3 Hz), 132.1 (d, ^3^*J*_CF_* =* 8.3 Hz), 132.3 (d, ^3^*J*_CF_* =* 8.0 Hz), 132.5, 134.6 (d, ^4^*J*_CF_ = 3.5 Hz), 135.8 (d, ^4^*J*_CF_ = 3.5 Hz), 139.2, 141.1, 147.0, 147.8, 152.1, 162.7 (d, ^1^*J*_CF_* =* 245.9 Hz), 162.9 (d, ^1^*J*_CF_* =* 247.1 Hz), 163.3 (d, ^1^*J*_CF_* =* 249.0 Hz), 191.2; *m/z* 440 (100, MH^+^); HRMS (ES): MH^+^, found 440.1257. C_28_H_17_NOF_3_^+^ requires 440.1262.

*4,6,8-Tris(4-methoxyphenyl)quinoline-3-carbaldehyde* (**2c**). Yellow solid (0.480 g, 60%), m.p. 218–220 °C (ethanol); *R*_f_ (10% ethyl acetate–hexane) 0.45; ν_max_ (ATR) 684, 745, 774, 829, 1029, 1176, 1244, 1379, 1412, 1486, 1513, 1569, 1604, 1676 cm^−1^; δ_H_ (CDCl_3_) 3.84 (s, 3H), 3.90 (s, 3H), 3.94 (s, 3H), 6.97 (d, *J =* 8.7 Hz, 2H), 7.07 (d, *J =* 8.4 Hz, 2H), 7.12 (d, *J =* 8.4 Hz, 2H), 7.40 (d, *J =* 8.4 Hz, 2H), 7.53 (d, *J =* 8.7 Hz, 2H), 7.69 (d, *J =* 8.4 Hz, 2H), 7.83 (d, *J =* 2.1 Hz, 1H), 8.06 (d, *J =* 2.1 Hz, 1H), 9.39 (s, 1H), 9.98 (s, 1H); δ_C_ (CDCl_3_) 55.3, 55.4, 55.5, 113.7, 114.1, 114.4, 123.3, 124.8, 125.6, 127.8, 128.5, 131.4, 131.8 (2×C), 132.1, 132.4, 139.4, 141.4, 147.0, 147.4, 153.2, 159.3, 159.7, 160.3, 192.1; *m/z* 476 (100, MH^+^); HRMS (ES): MH^+^, found 476.1871. C_31_H_26_NO_4_^+^ requires 476.1862.

*4,6,8-Tris(2-phenylethenyl)quinoline-3-carbaldehyde* (**2d**). Yellow solid (0.400 g, 60%), m.p. 199–200 °C (ethanol); *R*_f_ (10% ethyl acetate–hexane) 0.23; ν_max_ (ATR) 690, 752, 956, 973, 1226, 1449, 1566, 1684 cm^−1^; δ_H_ (CDCl_3_) 7.28–7.33 (m, 4H), 7.37–7.51 (m, 8H), 7.59 (d, *J =* 7.2 Hz, 2H), 7.67 (d, *J =* 7.2 Hz, 2H), 7.71 (d, *J =* 7.2 Hz, 2H), 7.77 (d, *J =* 16.2 Hz, 1H), 8.07 (d, *J =* 1.5 Hz, 1H), 8.38 (d, *J =* 1.5 Hz, 1H), 8.49 (d, *J =* 16.5 Hz, 1H), 9.31 (s, 1H), 10.43 (s, 1H); δ_C_ (CDCl_3_) 119.3, 123.0, 124.1, 124.6, 125.9, 126.6, 126.8, 127.1, 127.3, 127.5, 128.0, 128.2, 128.7, 128.8, 129.1, 129.5, 130.7, 131.2, 135.5, 135.9, 136.6, 136.7, 137.3, 142.2, 146.8, 147.6, 148.5, 191.4; *m/z* 464 (100, MH^+^); HRMS (ES): MH^+^, found 464.2016. C_34_H_26_NO^+^ requires 464.2014.

*4,6,8-Tris[2-(4-fluorophenyl)ethenyl]quinoline-3-carbaldehyde* (**2e**). Yellow solid (0.490 g, 67%), m.p. 224–225 °C (ethanol); *R*_f_ (10% ethyl acetate–hexane) 0.58; ν_max_ (ATR) 751, 818, 961, 976, 1157, 1221, 1507, 1597, 1677 cm^−1^; δ_H_ (CDCl_3_) 6.85 (d, *J =* 16.2 Hz, 1H), 7.05–7.27 (m, 7H), 7.37 (d, *J =* 16.5 Hz, 2H), 7.56 (t, *J =* 7.5 Hz, 2H), 7.62–7.72 (m, *5*H), 8.05 (s, 1H), 8.35 (s, 1H), 8.39 (d, *J =* 16.5 Hz, 1H), 9.30 (s, 1H), 10.42 (s, 1H); δ_C_ (CDCl_3_) 115.7 (d, ^2^*J*_CF_* =* 21.7 Hz), 115.8 (d, ^2^*J*_CF_* =* 21.7 Hz), 116.2 (d, ^2^*J*_CF_* =* 21.9 Hz), 119.1, 123.0, 124.0, 124.7, 126.0, 126.7, 127.4, 128.3 (d, ^3^*J*_CF_* =* 8.0 Hz), 128.6 (d, ^3^*J*_CF_* =* 8.0 Hz), 129.1 (d, ^3^*J*_CF_* =* 8.3 Hz), 129.8, 130.2, 131.8 (d, ^4^*J*_CF_* =* 3.2 Hz), 132.8 (d, ^4^*J*_CF_* =* 3.4 Hz), 133.5 (d, ^4^*J*_CF_* =* 3.2 Hz), 136.0, 136.9, 141.0, 147.0, 147.9, 148.4, 162.6 (d, ^1^*J*_CF_* =* 246.5 Hz), 162.7 (d, ^1^*J*_CF_* =* 247.0 Hz), 163.4 (d, ^1^*J*_CF_* =* 249.0 Hz), 191.4; *m/z* 518 (100, MH^+^); HRMS (ES): MH^+^, found 518.1737. C_34_H_23_NF_3_O^+^ requires 518.1732.

*4,6,8-Tris[2-(4-methoxyphenyl)ethenyl]quinoline-3-carbaldehyde* (**2f**). Yellow solid (0.490 g, 62%), m.p. 246–248 °C (ethanol); *R*_f_ (10% ethyl acetate–hexane) 0.18; ν_max_ (ATR) 820, 968, 1027, 1172, 1244, 1509, 1599, 1674 cm^−1^; δ_H_ (CDCl_3_) 3.84 (s, 3H), 3.85 (s, 3H), 3.88 (s, 3H), 6.81 (d, *J =* 16.8 Hz, 1H), 6.87–6.95 (m, 4H), 6.99 (d, *J =* 8.5 Hz, 2H), 7.13 (d, *J =* 16.2 Hz, 1H), 7.24–7.37 (m, 3H), 7.52 (d, *J =* 8.5 Hz, 2H), 7.58–7.67 (m, 4H), 8.01 (s, 1H), 8.31, (s, 1H), 8.34 (d, *J =* 16.2 Hz, 1H), 9.27 (s, 1H), 10.39 (s, 1H); δ_C_ (CDCl_3_) 55.3 (2xC), 55.4, 114.1, 144.2, 114.4, 117.0, 122.1, 124.3, 125.6, 125.9, 126.8, 127.4, 128.0, 128.4, 128.5, 128.8, 129.6, 130.2, 130.4, 136.3, 130.7, 136.3, 137.1, 142.0, 146.7, 147.5, 148.9, 159.5, 159.7, 160.7, 191.8; *m/z* 554 (100, MH^+^); HRMS (ES): MH^+^, found 554.2330. C_37_H_30_NO_4_^+^ requires 554.2331.

### 3.3. Typical Procedure for the Synthesis of 4,6,8-Triaryl-N-[(quinolin-3-yl)methylene]-3-benzene-amines **3*a***–***c***

A stirred mixture of **2a** (1 equiv.) and 4-fluorophenylaniline (3 equiv.) in dioxane (50 mL per mmol of **1**) was heated under Dean-Stark conditions for 3 h. The solvent was evaporated and the product was passed through a column of silica gel to afford **3**. The following products were prepared in this fashion:

*3-(4-Fluorophenyl)amino-4,6,8-(triphenyl)-N-(quinolin-3-yl)methylene* (**3a**). Off-white solid (0.280 g, 76%), m.p. 248–249 °C (EtOH); *R*_f_ (10% ethyl acetate–hexane) 0.72; ν_max_ (ATR) 600, 766, 1199, 1229, 1481, 1499, 1609 cm^−1^; δ_H_ (CDCl_3_) 7.23–7.35 (m, 4H), 7.58–7.73 (m. 6H), 7.77–7.88 (m, 7H), 8.02 (s, 2H), 8.04 (s, 1H), 8.28 (d, *J =* 3.0 Hz, 1H), 8.56 (s, 1H). 10.04 (s, 1H); δ_C_ (CDCl_3_) 115.8 (d, ^2^*J*_CF_* =* 22.5 Hz), 122.5 (d, ^3^*J*_CF_* =* 8.3 Hz), 124.1, 126.0, 127.5, 127.6, 127.8, 128.1, 128.7, 128.8, 128.9, 130.2, 130.3, 130.8, 131.2, 134.5, 139.4 (d, ^3^*J*_CF_* =* 2.6 Hz), 140.3, 141.7, 145.9, 147.6, 148.8, 149.7, 156.9, 164.4 (d, ^1^*J*_CF_* =* 243.9 Hz), 207.0; *m/z* 479 (100, MH^+^); HRMS (ES): MH^+^, found 479.1923. C_34_H_24_N_2_F^+^ requires 479.1924.

*4,6,8-Tris(4-fluorophenyl)-3-(4-fluorophenyl)amino-N-(quinolin-3-yl)methylene* (**3b**). Yellow solid (0.191 g, 78%), m.p. 253–254 °C (EtOH); *R*_f_ (10% ethyl acetate–hexane) 0.72; ν_max_ (ATR) 806, 830, 1158, 1226, 1487, 1499, 1511, 1604 cm^−1^; δ_H_ (CDCl_3_) 7.03 (t, *J =* 8.7 Hz, 2H), 7.08 (t, *J =* 8.7 Hz, 2H), 7.13 (t, *J =* 8.7 Hz, 2H), 7.23 (t, *J =* 8.7 Hz, 2H), 7.31 (t, *J =* 8.7 Hz, 2H) 7.43 (t, *J =* 8.7 Hz, 2H), 7.53 (t, *J =* 8.7 Hz, 2H), 7.65 (d, *J =* 2.1 Hz, 1H), 7.73 (t, *J =* 8.7 Hz,. 2H), 7.95 (d, *J =* 2.1 Hz, 1H), 8.30 (s, 1H), 9.77 (s, 1H); δ_C_ (CDCl_3_) 115.1 (d, ^2^*J*_CF_* =* 21.3 Hz), 115.9 (d, ^2^*J*_CF_* =* 21.3 Hz), 116.0 (d, ^2^*J*_CF_* =* 21.4 Hz), 116.1 (d, ^2^*J*_CF_* =* 21.4 Hz), 122.5 (d, ^3^*J*_CF_* =* 8.3 Hz), 123.6, 126.4, 127.7, 129.0 (d, ^3^*J*_CF_* =* 8.0 Hz), 130.2 (d, ^4^*J*_CF_* =* 3.7 Hz), 130.9, 131.9 (d, ^3^*J*_CF_* =* 8.0 Hz), 132.3 (d, ^3^*J*_CF_* =* 8.0 Hz), 135.0 (d, ^4^*J*_CF_* =* 3.4 Hz), 136.1 (d, ^4^*J*_CF_* =* 3.2 Hz), 138.6, 140.8, 145.7, 147.4, 148.4, 148.9, 156.2, 161.6 (d, ^1^*J*_CF_* =* 244.4 Hz), 162.6 (d, ^1^*J*_CF_* =* 245.6 Hz), 162.8 (d, ^1^*J*_CF_* =* 246.7 Hz), 163.0 (d, ^1^*J*_CF_* =* 241.3 Hz); *m/z* 533 (100, MH^+^); HRMS (ES): MH^+^, found 533.1645. C_34_H_21_N_2_F_4_^+^ requires 533.1641.

*3-(4-Fluorophenyl)amino-4,6,8-tris(4-methoxyphenyl)-N-(quinolin-3-yl)methylene* (**3c**). Yellow solid (0.170 g, 71%), mp. 217–218 °C (EtOH); *R*_f_ (10% ethyl acetate–hexane) 0.11; ν_max_ (ATR) 808, 823, 1036, 1174, 1246, 1485, 1498, 1514, 1607 cm^−1^; δ_H_ (CDCl_3_) 3.84 (s, 3H), 3.91 (s, 3H), 3.93 (s, 3H), 6.97 (d, *J =* 7.8 Hz, 2H), 7.04 (d, *J =* 7.8 Hz, 2H), 7.06–7.12 (m, 6H), 7.36 (d, *J =* 7.8 Hz, 2H), 7.53 (d, *J =* 7.8 Hz, 2H), 7.72 (d, *J =* 7.8 Hz, 2H), 7.74 (s, 1H), 7.98 (s, 1H), 8.35 (s, 1H), 9.73 (s, 1H); δ_C_ (CDCl_3_) 55.4 (2×C), 55.4, 113.6, 114.1, 114.3, 115.8 (d, ^2^*J*_CF_* =* 22.5 Hz), 122.5 (d, ^3^*J*_CF_* =* 8.3 Hz), 122.9, 126.2, 126.5, 128.0, 128.5, 130.5, 131.6, 131.8, 131.9, 132.8, 138.9, 141.2, 145.7, 147.8 (d, ^4^*J*_CF_* =* 2.9 Hz), 148.4, 149.5, 157.4, 159.2, 159.5, 159.8, 161.4 (d, ^4^*J*_CF_* =* 243.6 Hz); *m/z* 569 (100, MH^+^); HRMS (ES): MH^+^, found 569.2238. C_37_H_30_N_2_FO_3_^+^ requires 569.2240.

### 3.4. Typical Procedure for the Synthesis of 4,6,8-Triarylquinoline-3-methanol **4**

A stirred suspension of **2a** (1 equiv.) in ethanol (20 mL per mmol of **2a**) was treated with sodium borohydride (2 equiv.) at room temperature. The mixture was heated under reflux for 1 h and then quenched with an ice-cold water. The product was extracted into chloroform and the combined organic phases were dried over MgSO_4_, filtered and then evaporated under reduced pressure. The residue was purified by column chromatography to afford **4**. The following compounds were prepared in this fashion:

*4,6,8-Triphenylquinoline-3-methanol* (**4a**). Yellow solid (0.151 g, 75%), m.p. 169–170 °C (EtOH); *R*_f_ (10% ethyl acetate–hexane) 0.13; ν_max_ (ATR) 688, 761, 921, 1074, 1385, 1443, 1482, 1578, 1600, 1612 cm^−1^; δ_H_ (CDCl_3_) 4.62 (d, *J =* 5.7 Hz, 2H), 7.31–7.47 (m, 6H), 7.49–7.59 (m. 7H), 7.66 (d, *J =* 2.1 Hz, 1H), 7.73–7.76 (m, 2H), 7.97 (d, *J =* 2.1 Hz, 1H), 9.10 (s, 1H); δ_C_ (CDCl_3_) 61.3, 123.7, 127.4, 127.5, 127.7, 127.9, 129.1, 128.4, 128.7, 128.9, 129.4, 129.9, 130.5, 130.7, 135.7, 138.9, 139.7, 140.5, 141.4, 145.0, 146.8, 150.8; *m/z* 388 (100, MH^+^); HRMS (ES): MH^+^, found 388.1689. C_28_H_21_NO^+^ requires 388.1701.

*4,6,8-Tris(4-fluorophenyl)quinoline-3-methanol* (**4b**). Yellow solid (0.159 g, 80%), m.p. 188–190 °C (EtOH); *R*_f_ (10% ethyl acetate–hexane) 0.14; ν_max_ (ATR) 809, 834, 1080, 1155, 1221, 1383, 1490, 1512, 1605 cm^−1^; δ_H_ (CDCl_3_) 4.59 (s, 2H), 7.11 (t, *J =* 8.7 Hz, 2H), 7.29 (t, *J =* 8.7 Hz, 2H), 7.26 (t, *J =* 8.7 Hz, 2H), 7.35 (t, *J =* 8.7 Hz, 2H), 7,51 (t, *J =* 8.7 Hz, 2H), 7.68 (d, *J =* 2.4 Hz, 1H), 7.70 (t, *J =* 8.7 Hz, 2H), 7.88 (d, *J =* 2.4 Hz, 1H), 9.09 (s, 1H); δ_C_ (CDCl_3_) 61.1, 115.1 (d, ^2^*J*_CF_* =* 21.4 Hz), 115.9 (d, ^2^*J*_CF_* =* 21.3 Hz, 2×C), 123.3, 128.0, 129.0 (d, ^3^*J*_CF_* =* 8.0 Hz), 129.7, 130.9, 131.2 (d, ^3^*J*_CF_* =* 8.0 Hz), 131.3 (d, ^4^*J*_CF_* =* 3.2 Hz), 132.2 (d, ^3^*J*_CF_* =* 7.7 Hz), 135.3 (d, ^4^*J*_CF_* =* 3.2 Hz), 136.3 (d, ^4^*J*_CF_* =* 3.2 Hz), 138.1, 140.9, 144.8, 145.7, 150.8, 162.5 (d, ^1^*J*_CF_* =* 245.3 Hz), 162.7 (d, ^1^*J*_CF_* =* 246.8 Hz, 2×C); *m/z* 442 (100, MH^+^); HRMS (ES): MH^+^, found 442.1425. C_28_H_19_NF_3_O^+^ requires 442.1419.

*4,6,8-Tris(4-methoxyphenyl)quinoline-3-methanol* (**4c**). Yellow solid (0.152 g, 70%), m.p. 135–136 °C (EtOH); *R*_f_ (10% ethyl acetate–hexane) 0.04; ν_max_ (ATR) 807, 823, 1036, 1246, 1385, 1498, 1514, 1608 cm^−1^; δ_H_ (CDCl_3_) 3.82 (s, 3H), 3.88 (s, 3H), 3.91 (s, 3H), 4.60 (s, 2H), 7.11 (t, *J =* 8.7 Hz, 2H), 6.74 (s, 1H), 7.05 (t, *J =* 8.7 Hz, 2H), 7.06 (d, *J =* 8.7 Hz, 2H), 7.27 (d, *J =* 8.7 Hz, 2H), 7.50 (d, *J =* 8.7 Hz, 2H), 7.62 (d, *J =* 2.1 Hz, 1H), 7.68 (d, *J =* 8.7 Hz, 2H), 7.90 (d, *J =* 2.1 Hz, 1H), 9.05 (s, 1H); δ_C_ (CDCl_3_) 55.3, 55.4 (2×C), 61.4, 113.6, 114.1, 114.3, 114.7, 116.0, 122.6, 127.7, 128.4, 128,5, 129.3, 130.7, 130.8, 131.7, 132.1, 133.0, 138.4, 140.8, 144.8, 146.5, 150.4, 159.1, 159.4, 159.5; *m/z* 478 (100, MH^+^); HRMS (ES): MH^+^, found 478.2021. C_31_H_28_NO_4_^+^ requires 478.2018.

## 4. Conclusions

Elaboration of the 6,8-dibromo-4-chloroquinoline-3-carbaldehyde scaffold via Suzuki-Miyauri cross-coupling with aryl- and arylvinylboronic acids afforded polysubstituted quinoline derivatives with potential photophysical and biological properties. The absorption and fluorescent properties of the 4,6,8-triarylquinoline-3-carbaldehydes as well as 4,6,8-triaryl-3-(4-aryl)amino)-*N*-(quinolin-3-yl)-methylenes and 4,6,8-triarylquinoline-3-methanols prepared in this investigation showed strong correlation with the substituent on the aryl, styryl and amino-*N*-methylene groups as well as the effect of the solvent. The polyaryl quinoline-3-carbaldehydes **2a**–**f**, have better fluorescence properties in the three solvent tested, except for **2a** which showed poor fluorescence in DMF. The solvent-dependent emission characteristics of these polyarylquinoline-3-carbaldehydes may result from the dipolar interaction with the polar solvents thus suggesting the ICT character of the emission state. The 4,6,8-triarylquinoline-3-methanols **4a**–**c** and the 4,6,8-triaryl-3-(4-aryl)amino)-*N*-(quinolin-3-yl)methylenes **3a**–**c**, on the other hand, exhibit reduced emission intensity compared to the corresponding precursors. This effect may be tentatively attributed to non-radiative decay to the lowest vibrational energy level of the excited state. The compounds described in this investigation represent suitable candidates for further synthesis of metal complexes, e.g., with iridium, palladium, platinum, *etc.* This is because polyarylsubstituted quinolines have been metalated with iridium to form cyclometalated iridium complexes with potential application in organic light-emitting diodes (OLEDs) [9,10,12] or novel red-emitting electrophosphorescent devices [2].
